# Neuroimaging markers of dual impairment in cognition and physical performance following stroke: The Nor-COAST study

**DOI:** 10.3389/fnagi.2022.1037936

**Published:** 2022-12-06

**Authors:** Marte Stine Einstad, Till Schellhorn, Pernille Thingstad, Stian Lydersen, Eva Birgitte Aamodt, Mona Kristiansen Beyer, Ingvild Saltvedt, Torunn Askim

**Affiliations:** ^1^Department of Neuromedicine and Movement Science, Faculty of Medicine and Health Sciences, NTNU-Norwegian University of Science and Technology, Trondheim, Norway; ^2^Institute of Clinical Medicine, University of Oslo, Oslo, Norway; ^3^Division of Radiology and Nuclear Medicine, Oslo University Hospital, Oslo, Norway; ^4^Department of Mental Health, Faculty of Medicine and Health Sciences, NTNU-Norwegian University of Science and Technology, Trondheim, Norway; ^5^Department of Geriatric Medicine, St. Olavs hospital, Trondheim University Hospital, Trondheim, Norway; ^6^Stroke Unit, Department of Internal Medicine, St. Olavs Hospital, Trondheim University Hospital, Trondheim, Norway

**Keywords:** stroke, cognition, physical performance, neuroimaging, MRI

## Abstract

**Background:**

Cognitive decline and decline in physical performance are common after stroke. Concurrent impairments in the two domains are reported to give increased risk of dementia and functional decline. The concept of dual impairment of physical performance and cognition after stroke is poorly investigated. Clinically accessible imaging markers of stroke and pre-existing brain pathology might help identify patients at risk.

**Objective:**

The primary aim of this study was to investigate to which extent pre-stroke cerebral pathology was associated with dual impairment in cognition and physical performance at time of stroke. Secondary aims were to examine whether white matter hyperintensities, medial temporal lobe atrophy, and stroke lesion volume and location were associated with dual impairment.

**Methods:**

Participants from the Norwegian Cognitive Impairment After Stroke (Nor-COAST) study with available MRI data at baseline were included in this cross-sectional study. Logistic regression analyses were conducted, with impairment status (no impairment, impaired cognition, impaired physical performance, and dual impairment) as the dependent variable and MRI markers as covariates. Pre-existing brain pathologies were classified into neurodegenerative, cerebrovascular, or mixed pathology. In addition, white matter hyperintensities and medial temporal lobe atrophy were included as independent covariates. Stroke volume and location were also ascertained from study-specific MRI scans.

**Results:**

Participants’ (*n* = 348) mean (SD) age was 72.3 (11.3) years; 148 (42.5%) were women. Participants with dual impairment (*n* = 99) were significantly older, had experienced a more severe stroke, and had a higher comorbidity burden and poorer pre-stroke function. Stroke lesion volume (odds ratio 1.03, 95%, confidence interval 1.00 to 1.05, *p* = 0.035), but not stroke location or pre-existing brain pathology, was associated with dual impairment, after adjusting for age and sex.

**Conclusion:**

In this large cohort of stroke survivors having suffered mainly mild to moderate stroke, stroke lesion volume—but not pre-existing brain pathology—was associated with dual impairment early after stroke, confirming the role of stroke severity in functional decline.

## Introduction

The emerging concept of “dual decliners” describes individuals with dual impairment of physical performance and cognition; these have been reported to be associated with a higher risk of progression to dementia and hospitalization in community-dwelling older adults (Tian et al., 2020). This might be regarded as a continuation of the motoric cognitive risk syndrome (MCR), a predementia syndrome combining subjective cognitive complaints and slow gait speed (Verghese et al., 2013). MCR has shown to be associated with lower gray matter volume, which suggests a closer relationship with neurodegenerative dementias (Sekhon et al., 2019; Beauchet et al., 2020). However, MCR has also shown to be associated with cerebrovascular changes (Wang et al., 2016). This inconsistency in the literature indicates a need for further investigation.

In two different cohorts of older people characterized by a high prevalence of comorbidities, including cerebrovascular disease, it is shown that dual impairments were associated with pathological changes in brain areas like the thalamus, precuneus, and superior frontal gyrus (Tian et al., 2021) and increased risk of decline in instrumental activities of daily living (Jokinen et al., 2021). The concept of dual impairment has been sparsely investigated in stroke survivor populations who are at an increased risk of dementia. However, a recent study reported a prevalence of 20% of dual impairment in the study cohort 3 months after suffering a mild or moderate stroke (Einstad et al., 2021). Knowledge about how different types of brain pathology are associated with impaired cognition, impaired physical performance, and dual impairment might shed light on potential brain mechanisms underlying functional decline after stroke.

The observed impairments in cognition and physical performance after a stroke may be a symptom of the focal signs from the stroke lesion itself as well as of pre-existing dispersed brain pathology (Zhang et al., 2017; Puy et al., 2018; Khan et al., 2019). Processes related to both the stroke lesion and pre-existing cerebral abnormalities might contribute to lower brain resilience, placing individuals at risk for dual impairment (Poggesi et al., 2014; Lee et al., 2017).

Existing findings in stroke patients have reported that other cerebral abnormalities are commonly present at the time of the stroke incident, including white matter hyperintensities (WMH), microbleeds, medial temporal lobe atrophy (MTA), and increased ventricular volume (Schellhorn et al., 2021a). Schellhorn and colleagues classified patients into groups primarily characterized by pre-existing neurodegeneration, cerebrovascular disease, or mixed pathology. Patients in the cerebrovascular disease or mixed pathology groups were significantly more likely to have suffered from pre-stroke cognitive impairments (Schellhorn et al., 2021a). In addition, the volume, and the location of the stroke lesion itself have been reported as significant predictors of post-stroke functional outcome (Zaidi et al., 2012; Jones et al., 2016; Puy et al., 2018). More specifically, the thalamus and the middle frontal gyrus have been identified as strategic stroke locations for cognition, and the insula, the putamen, and the external capsule have been associated with changes in physical performance (Jones et al., 2016; Puy et al., 2018). It seems that the infarct volume is to a greater extent related to post-stroke physical performance, while WMH (interpreted as an expression of cerebral small vessel disease) are associated with cognitive impairments (Auriat et al., 2019; Georgakis et al., 2019; Khan et al., 2019). Furthermore, findings from Hawe et al. revealed that a larger burden of WMH worsens the impact of stroke lesion volume, especially on cognition (Hawe et al., 2018).

These findings suggest an interplay between stroke lesion characteristics and pre-existing pathology that should be further explored. However, the association between pre-existing brain pathology and post-stroke dual impairment of cognition and physical performance is yet to be investigated. Despite conflicting evidence within the field of dual impairment, it is vital to increase our understanding of the associations between pre-existing and stroke-related brain pathology and the combination of cognitive and motor impairments, in order to make future progress within post-stroke neurorehabilitation.

The overall aim of the present study was to explore the relationship between pre-and post-stroke cerebral MRI markers obtained from MRI scans performed within the first week after stroke, and the separate and combined outcomes of cognition and physical performance impairments in the acute phase after stroke. The primary aim was to investigate the extent to which pre-stroke cerebral pathology (neurodegeneration, cerebrovascular disease, or mixed pathology) was associated with post-stroke dual impairments in cognition and physical function. The secondary aims were to examine the associations between WMH, MTA, and stroke lesion characteristics (lesion volume and location) at time of stroke and post-stroke impairment groups.

## Materials and methods

### Study design and participants

The present study was a cross-sectional study with baseline data from the Norwegian Cognitive Impairment After Stroke (Nor-COAST) study, a multicenter prospective cohort study that includes participants recruited from three university hospitals and two local hospitals in Norway between May 2015 and March 2017 (Thingstad et al., 2018). Inclusion criteria were: diagnosed with stroke according to the WHO criteria (WHO, 1988), symptom onset within 1 week before admission, being over 18 years old, fluency in a Scandinavian language, and living in the catchment area of the participating hospitals. Patients with less than 3 months of expected survival were excluded from the study. More details about the Nor-COAST protocol are given in Thingstad et al. (2018).

A subsample of the Nor-COAST participants was included in an MRI sub-study; for this sub-study, the inclusion criterion was the ability to cooperate during an MRI scan, and exclusion criteria were severe functional impairment (modified Rankin Scale score > 3), medical contraindications for MRI (e.g., claustrophobia or pacemaker), and/or refusal to participate in the MRI sub-study. In order to be included in the present study, participants needed to have available scores on the Montreal Cognitive Assessment (MoCA) and the Short Physical Performance Battery (SPPB) (see below) at baseline.

### MRI acquisition

Participants underwent a study-specific MRI protocol at one of the five participating hospitals 2–7 days after onset of stroke. The study protocol included a 3D T1-weighted sequence, axial T2, 3D Fluid attenuated inversion recovery (FLAIR), diffusion weighted imaging (DWI), and susceptibility weighted imaging (SWI). Further details about the MRI study protocol are described in Schellhorn et al. (2021a) In addition, 63 participants who were not included in the MRI sub-study had available clinical MRIs that were suitable for visual rating, and these participants were included in the analyses. The clinical MRI sequences were acquired with different sequence parameters than the study-specific MRIs but were suitable for visual rating.

### Image analysis

#### Visually rated variables: Pre-stroke

Pre-existing pathology markers were visually scored according to established criteria. Standards for reporting Vascular Changes on Neuroimaging (STRIVE) recommendations were applied to rate small vessel disease features (Wardlaw et al., 2013); for neurodegenerative disease, validated visual rating scales were used (Ferreira et al., 2015). WMH of presumed vascular origin were classified using the widely used Fazekas scale (Fazekas et al., 1987) and categorized as normal or pathological by combining score and age. The presence of lacunes of presumed vascular origin assessed on the 3D FLAIR was always regarded as pathological (Donnan and Norrving, 2008). Microbleeds were classified as present when ≥2 hypointense lesions were found on SWI (Cordonnier et al., 2009; Poels et al., 2011), and old infarcts were classified as present when there was parenchymal defect with significant loss of volume without corresponding diffusion restriction. MTA was assessed according to the established MTA scale (Scheltens et al., 1992) and was scored as normal or pathological according to reference values adjusted for age (Ferreira et al., 2015). Posterior atrophy was assessed with the posterior atrophy scale (Koedam et al., 2011), and a value of ≥2 was considered pathological in participants older than 95 years (Ferreira et al., 2015). Ventricular enlargement, which is considered an expression of global atrophy, was measured by the Evans Index (Evans, 1942) and was classified as normal or pathological according to sex-and age-dependent reference values (Brix et al., 2017).

Participants were sorted into four groups: (1) No pathological scores in visually rated brain MRI; (2) neurodegeneration (pathological markers of MTA, posterior atrophy, or ventricular enlargement, and no pathological markers of cerebrovascular disease); (3) cerebrovascular disease (pathological scoring of WMH, lacunes, or microbleeds, and no pathological markers of neurodegeneration); and (4) mixed pathology (presence of pathological scores of both neurodegeneration and cerebrovascular disease).

#### Stroke lesion volume and location: Post-stroke

Stroke volume lesion masks were created for participants with visible diffusion restrictions on DWI. Stroke lesion volume was specified as equivalent to the ischemic core, which represents the amount of irreversibly destroyed brain parenchyma, identified as diffusion restriction on the DWI sequence. The ITK “Insight Segmentation and Registration Toolkit-Snap” (ITK-Snap) snake tool (v. 3.8.0) (Yushkevich et al., 2006) was used to semi-automatically label acute infarcts in order to create lesion masks, which were created for all participants who had visible diffusion restriction on DWI. The masked stroke volume in mm^3^ was automatically measured by ITK-Snap and converted to milliliters (ml) after being exported to a comma-separated values (csv) file. Further details on stroke volume extraction in this sample are described in Aamodt et al. (2021).

The location of the acute stroke was identified using the coordinates of the stroke lesion masks to find the corresponding anatomical structure at the gyrus level in the Talairach brain atlases (Lancaster et al., 2000). Gyri, thalami, and nuclei were classified as gray matter, and sub-gyral and extra-nuclear spaces were classified as white matter; cerebellar structures were classified as cerebellum. In participants with multiple lesions in more than one region, we defined gray matter was set as the location if at least one lesion had this location, and white matter as the location if all lesions were situated in white matter.

### Participants’ characteristics

Baseline characteristics were retrieved from participants, proxies, and medical records during their hospital stay. Clinical assessments were performed by trained health care personnel according to a standardized manual before discharge or within a week after admission. TOAST (Trial of Org 10,172 in Acute Stroke Treatment) classification which categorizes ischemic stroke by etiology, was carried out by experienced stroke physicians (Adams et al., 1993). The National Institutes of Health Stroke Scale (NIHSS) (Brott et al., 1989) score at admission was used to measure stroke severity; the possible score range was 0–42, with higher scores indicating more severe stroke. Functional dependency was measured with the Modified Rankin Scale (mRS) (van Swieten et al., 1988), a scale (0–6) where a higher score indicates more dependency, and a score of 6 denotes death. Pre-stroke mRS score was assessed retrospectively while post-stroke mRS was assessed either at discharge or day 7 after symptom onset. Pre-stroke conditions—such as hypertension, diabetes mellitus, previous stroke, and atrial fibrillation—were evaluated as present based on medication prescribed before admission, in-hospital assessments, and medical records. A Charlson Comorbidity Index score was calculated based on participant information and medical records and was used as a descriptive measure to quantify comorbidity (Charlson et al., 1987). The index is based on 19 medical conditions weighted according to severity, and is categorize into three grades: mild, with scores of 1–2; moderate, with scores of 3–4; and severe, with scores ≥5. Frailty was measured by the Fried Frailty Index, a three-category ordinal scale defining participants as robust, pre-frail or frail based on the criteria weight loss, exhaustion, physical activity, gait speed and grip strength (Fried et al., 2001).

### Clinical assessments: Post-stroke

We assessed physical performance with the Short Physical Performance Battery (SPPB), a measure that consists of three tasks: four-meter preferred gait speed, balance, and five times sit-to-stand from chair. It uses four-point scales for each task for a sum score ranging from 0 to 12, with higher scores indicating better function (Guralnik et al., 1994, 1995). Global cognition was assessed with the Montreal Cognitive Assessment (MoCA), a 10-item test covering eight cognitive domains: short-term memory recall, visuospatial abilities, executive function, attention, concentration, working memory, language, and orientation. Possible scores range from 0 to 30, with higher scores indicating better cognition (Nasreddine et al., 2005). A cut-off for impairment was set at <10 points for the SPPB (Guralnik et al., 1995) and < 24 points for the MoCA (Borland et al., 2017). Participants were classified as having: (1) no impairments, (2) impaired cognition, (3) impaired physical performance, or (4) dual impairment.

### Ethical considerations

The present study was carried out according to the Declaration of Helsinki. Participation was voluntary and based on written informed consent from the participant or, in cases where participants were not able to give consent, by their proxy. The study was approved by the Regional Committee for Medical and Health Research Ethics (REC Central 194265) and registered at ClinicalTrials.gov (NCT02650531).

### Statistics

MRI scans were carried out within a week after admission to hospital, patient interviews and clinical assessments were done before discharge or at the latest 1 week after admission. Demographic and clinical characteristics were summarized using means and standard deviations (SD) for continuous and ordinal variables and frequencies and percentages for dichotomous variables. Mean imputation was carried out in cases with single items missing on the MoCA and NIHSS. Cases with more than 50% missing items were excluded from analyses (*n* = 1 for MoCA). Missing scores on the SPPB were not imputed, due to too few variables being part of the total score. Baseline characteristics were compared across impairment groups using the Pearson chi-square test for dichotomous and nominal categorical variables and the Kruskal–Wallis test for ordinal and continuous variables.

We used multinominal logistic regression models, with post-stroke impairment group (no impairment, impaired cognition, impaired physical function, and dual impairment) as the dependent variable and type of pre-stroke brain pathology (neurodegeneration, cerebrovascular disease, or mixed pathology, with normal as reference category), stroke volume, stroke location (right hemisphere and white matter as reference variables), WMH, and MTA as covariates. As few participants (*n* = 12) were classified with cerebellar lesion, participants with this location were not included in the regression analyses including stroke location as covariate. WMH and MTA were used as ordinal variables in the analyses. Analyses were carried out with and without adjustment for age and sex. Level of statistical significance was set at two-tailed *p* < 0.05 in the regression analyses; in the comparisons between clinical characteristics, value of ps between 0.01 and 0.05 should be interpreted with caution, due to multiple hypotheses. Data were analyzed using Stata/MP 17.

## Results

### Participant characteristics

**Figure 1:**
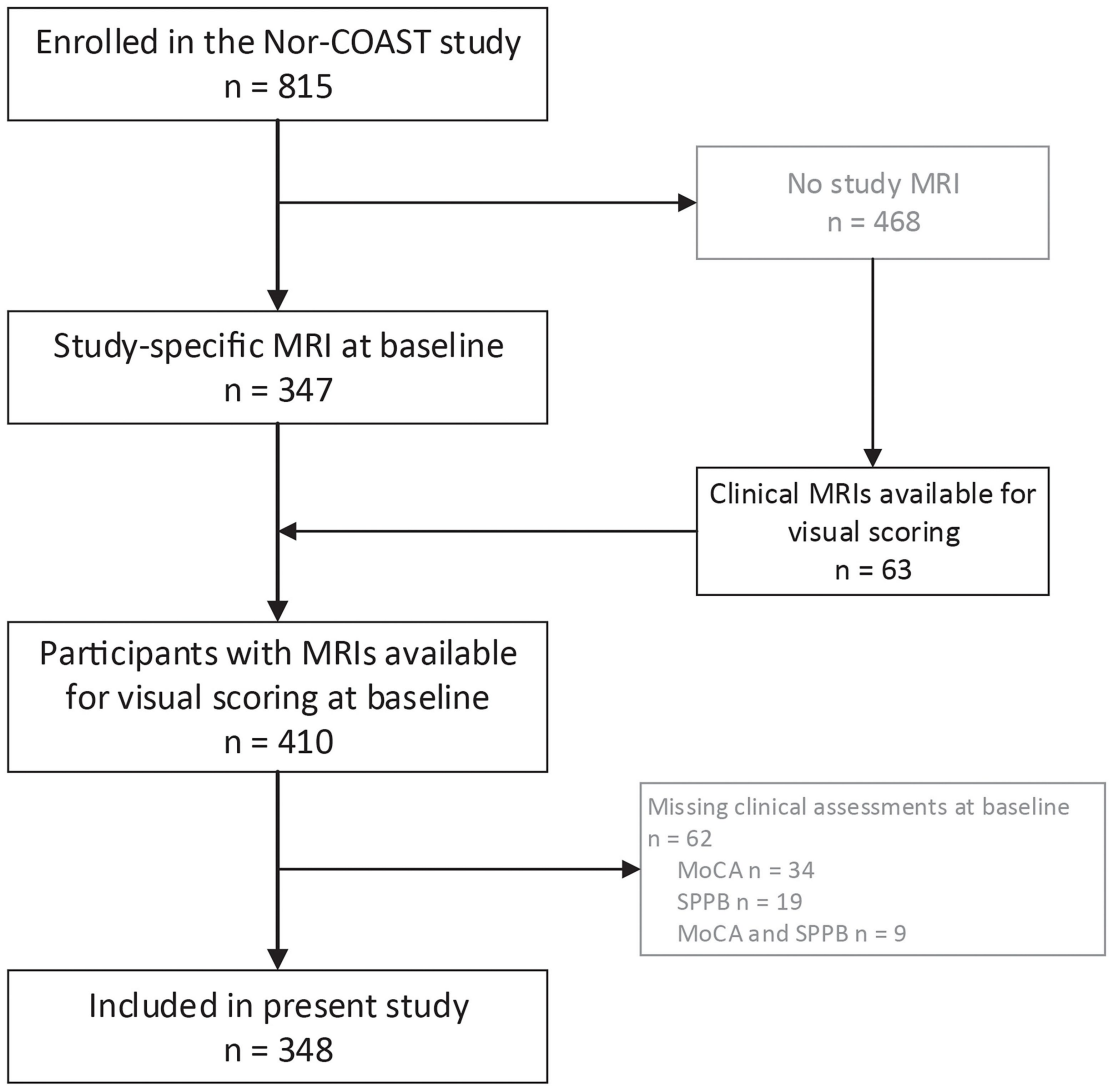
Flow chart on inclusion. MoCA-Montreal Cognitive Assessment; MRI-magnetic resonance imaging; Nor-COAST, Norwegian Cognitive Impairment After Stroke; SPPB-Short Physical Performance Battery.

Of the 815 participants included in Nor-COAST, 410 participants had MRIs available for visual scoring; of these, 348 had available clinical assessments and were included in the present study (Figure 1). Those who were excluded from the study sample of 348 participants were significantly older (mean (SD) age 74.4 (12.0) years versus 72.3 (11.3) years, *p* = 0.012), had poorer pre-stroke function (mean (SD) mRS score 1.1 (1.3) versus 0.8 (1.0), *p* < 0.001), and had suffered from more severe strokes (mean (SD) NIHSS score 5.4 (6.9) versus 3.6 (4.5), *p* < 0.001).

Of the included participants, 235 (43.2%) were women; 324 (93.9%) had suffered from an infarction, while 21 (6.1%) had suffered from cerebral hemorrhage. The mean (SD) MoCA score was 24.1 (4.9) points, and the mean (SD) SPPB score was 7.9 (3.7). One or more pathological pre-stroke imaging markers were found in 234 (67.2%) of the participants, and 82 (23.6%) had mixed pathology (Table 1).

**Table 1:** Neuroimaging characteristics.

	Total sample	No impairment	Impaired cognition	Impaired physical performance	Dual impairment
**Prestroke characteristics**					
Normal, *n* (%)	114 (32.8)	45 (38.5)	6 (22.2)	40 (38.1)	23 (23.2)
Vascular pathology, *n* (%)	100 (28.7)	27 (23.1)	9 (33.3)	31 (29.5)	33 (33.3)
Neurodegeneration, *n* (%)	52 (14.9)	19 (16.2)	7 (25.9)	12 (11.4)	14 (14.1)
Mixed pathology, *n* (%)	82 (23.6)	26 (22.2)	5 (18.5)	22 (21.0)	29 (29.3)
MTA, mean (SD)	1.2 (0.8)	1.1 (0.7)	1.4 (0.7)	1.1 (0.7)	1.4 (0.8)
MTA, median (IQR)	1 (0.5)	1 (0.5)	1.5 (0.5)	1 (0.5)	1.5 (0.5)
WMH, mean (SD)	1.8 (0.9)	1.5 (0.9)	1.9 (0.9)	1.8 (0.9)	2.2 (0.9)
WMH, median (IQR)	2.0 (1.0)	1.0 (1.0)	2.0 (1.0)	2.0 (1.0)	3.0 (1.0)
**Stroke-related characteristics**					
Stroke lesion volume (ml), mean (SD)^a^	6.9 (16.0)	4.7 (10.5)	4.6 (8.7)	6.4 (15.6)	10.1 (21.3)
Location^b^ *n* (%)					
Gray matter	107 (46.5)	36 (48.7)	11 (68.8)	26 (34.2)	34 (53.1)
White matter	111 (48.3)	33 (44.6)	5 (31.3)	45 (59.2)	28 (43.8)
Cerebellum	12 (5.2)	5 (6.8)	0 (0.0)	5 (6.6)	2 (3.1)
Left hemisphere, *n* (%)	101 (44.5)	29 (41.4)	8 (50.0)	34 (45.3)	30 (45.5)

IQR, interquartile range; MTA, medial temporal lobe atrophy (0–4); SD, standard deviation WMH, white matter hyperintensities (0–3).

Vascular pathology: Pathological WMH score or presence of old infarcts, lacunes or ≥ 2 microbleeds.

Neurodegeneration: Pathological MTA, posterior atrophy, or ventricular enlargement.

Mixed pathology: Presence of both vascular pathology and neurodegeneration.

a *n* = 288.

b *n* = 230.

### Post-stroke impairment groups

**Figure 2:**
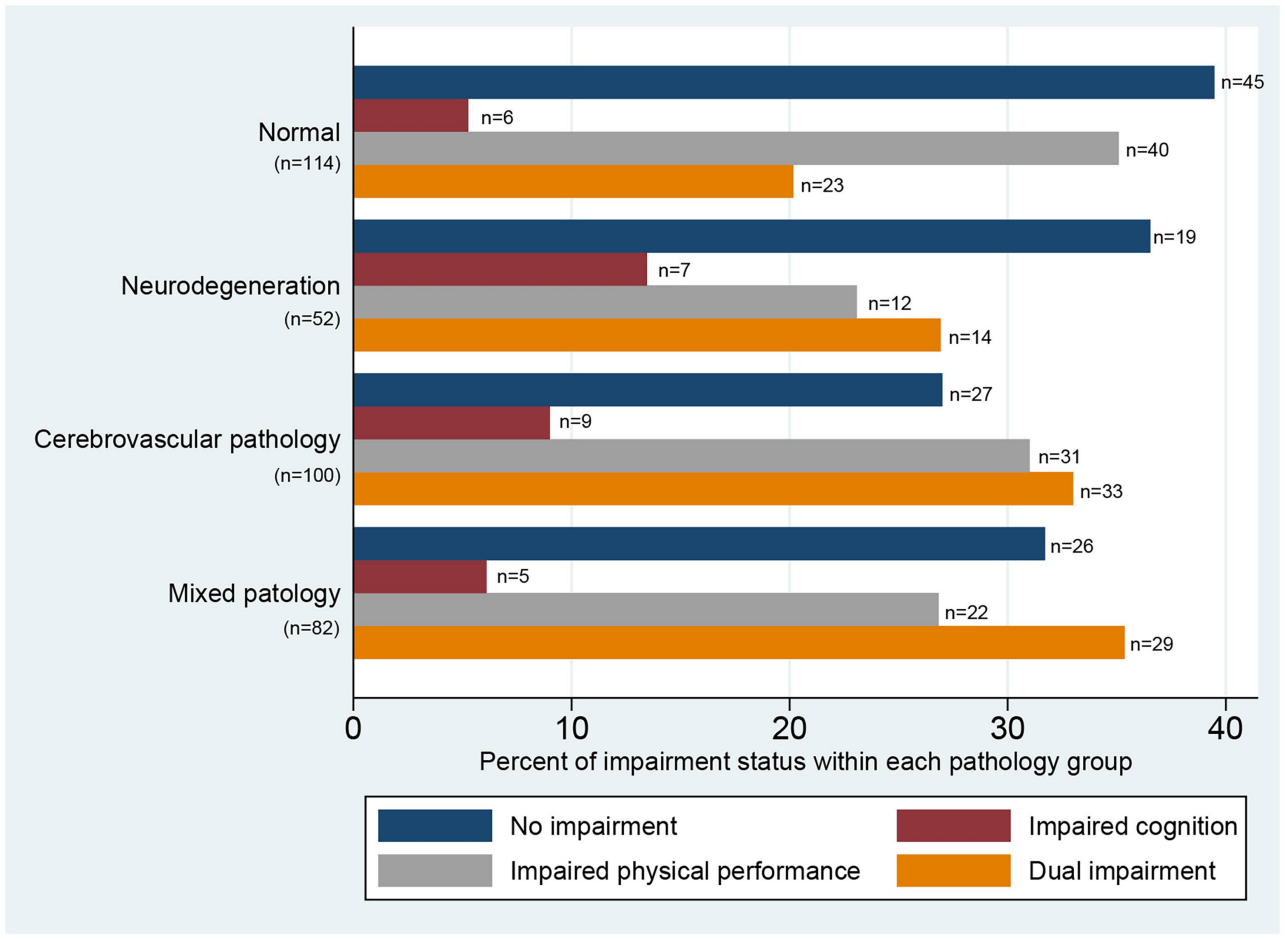
Distribution of impairment status within pathology groups illustrated by percentages within each pathology group. No impairment = Short Physical Performance Battery (SPPB) ≥10p and Montreal Cognitive Assessment (MoCA) ≥24p; impaired cognition = SPPB ≥10p and MoCA <24p; impaired physical performance = SPPB <10p and MoCA ≥24p; dual impairment = SPPB <10p and MoCA <24p. Vascular pathology: Pathological white matter hyperintensities score or presence of old infarcts, lacunes or ≥ 2 microbleeds. Neurodegeneration: Pathological medial temporal lobe atrophy, posterior atrophy, or ventricular enlargement. Mixed pathology: Presence of both vascular pathology and neurodegeneration.

Of the included participants, 117 (33.6%) had normal scores on both the MoCA and SPPB; 27 (7.8%) and 105 (30.2%) had reduced scores on only the MoCA or SPPB, respectively; and 99 (28.4%) had dual impairment, with reduced scores on both the MoCA and SPPB. Participants with dual impairment were significantly older, had fewer years of education, had more severe strokes (according to NIHSS scores), had more comorbidity, had a higher prevalence of hypertension and cardiovascular disease, and had higher pre-stroke mRS scores (Table 2). The distribution of impairments within the neuropathology groups are displayed in Figure 2.

**Table 2:** Baseline characteristics.

	Total sample	No impairment	Impaired cognition	Impaired physical performance	Dual impairment	*p*-Value
Participants, *n* (%)	348 (100)	117 (33.6)	27 (7.8)	105 (30.2)	99 (28.4)	
Age (8 years), mean (SD)	72.3 (11.3)	65.7 (11.0)	76.3 (8.1)	72.9 (9.9)	78.5 (9.7)	<0.001
Women, *n* (%)	148 (42.5)	46 (39.3)	6 (22.2)	50 (47.6)	46.5 (46.5)	0.079
Education (years), mean (SD)	12.3 (3.8)	13.7 (3.5)	12.3 (3.8)	12.6 (4.0)	10.4 (3.1)	<0.001
**Stroke characteristics**						
Hemorrhage	21 (6.1)	10 (8.6)	1 (3.7)	3 (2.9)	7 (7.1)	
TOAST^a^ classification, *n* (%)	*N* = 315	*N* = 102	*N* = 25	*N* = 99	*N* = 89	0.235
Large artery disease	34 (10.8)	10 (9.8)	1 (4.0)	10 (10.1)	13 (14.6)	
Cardial emboli	71 (22.5)	20 (19.6)	6 (24.0)	17 (17.2)	28 (31.5)	
Small vessel disease	82 (26.0)	28 (27.5)	6 (24.0)	31 (31.3)	17 (19.1)	
Other etiology	6 (1.9)	1 (1.0)	0 (0.0)	4 (4.0)	1 (1.1)	
Undetermined etiology	122 (38.7)	43 (42.2)	12 (48.0)	37 (37.4)	30 (33.7)	
NIHSS (admittance), mean (SD)	3.6 (4.5)	2.4 (2.6)	4.7 (8.2)	3.4 (3.7)	5.0 (5.0)	<0.001
**Comorbidity**						
Previous stroke or TIA, *n* (%)	80 (23.0)	19 (16.2)	5 (18.5)	24 (22.9)	32 (32.3)	0.042
Hypertension, *n* (%)	189 (54.3)	53 (45.3)	12 (44.4)	58 (55.2)	66 (66.7)	0.012
Diabetes mellitus, *n* (%)	74 (21.3)	18 (15.4)	4 (14.8)	23 (21.9)	29 (29.3)	0.074
Hypercholesterolemia, *n* (%)	166 (47.7)	53 (45.3)	10 (37.0)	59 (56.2)	44 (44.4)	0.175
Current cigarette smoking, *n* (%)	68 (19.5)	18 (15.4)	7 (25.9)	28 (26.7)	15 (15.2)	0.086
Atrial fibrillation, *n* (%)	77 (22.1)	21 (18.0)	5 (18.5)	22 (21.0)	29 (29.3)	0.219
Coronary heart disease, *n* (%)	57 (16.4)	14 (12.0)	2 (7.4)	14 (13.3)	27 (27.3)	0.006
Charlson Comorbidity index, mean (SD)	3.9 (1.9)	3.0 (1.7)	4.0 (1.8)	4.0 (1.7)	4.9 (1.8)	<0.001
Frail, prestroke, *n* (%)	45 (12.9)	1 (0.8)	15 (55.6)	17 (16.2)	27 (27.3)	<0.001
**Functional assessments**						
mRS, prestroke, mean (SD)	0.8 (1.0)	0.5 (0.7)	0.6 (0.8)	0.8 (0.9)	1.2 (1.2)	<0.001
mRS at discharge, mean (SD)	2.1 (1.3)	1.3 (0.8)	1.6(1.1)	2.3 (1.3)	2.8 (1.2)	<0.001
MoCA, mean (SD)	24.1 (4.9)	27.6 (1.8)	19.3 (3.9)	26.6 (1.7)	18.6 (3.8)	<0.001
SPPB, mean (SD)	7.9 (3.7)	11.4 (0.8)	10.8 (0.9)	6.1 (2.8)	4.8 (3.1)	<0.001

MoCA, Montreal Cognitive Assessment; mRS, modified Rankin Scale; NIHSS, National Institute of Health Stroke Scale; SD, standard deviation; SPPB, Short Physical Performance Battery; TIA, transient ischemic attack; TOAST, Trial of Org 10,172 in Acute Stroke Treatment

Pearson chi square test for dichotomous and nominal categorical variables and Kruskal–Wallis test for ordinal and continuous variables.

Frailty measured by Fried frailty index.

a *n* = 315, missing data from 33 participants.

### Results from regression analyses

Unadjusted binominal logistic regression analysis with MRI pathology group as a categorical variable showed that cerebrovascular pathology and mixed pathology were associated with dual impairment, with OR 2.39 (95% CI 1.17 to 4.89), *p* = 0.017, and OR 2.18 (95% CI 1.05 to 4.53) *p* = 0.036, respectively (Table 3). However, as displayed in Table 4, the associations declined and became nonsignificant after adjusting for age and sex.

**Table 3:** Multinominal logistic regression, unadjusted.

Covariate	Impaired cognition			Impaired physical performance			Dual impairment		
	OR	95% CI	*p*-value	OR	95% CI	*p*-value	OR	95% CI	*p*-value
**Pathology groups**^**a**^									
Neurodegeneration	2.76	0.82 to 9.31	0.101	0.71	0.31 to 1.64	0.425	1.44	0.61 to 3.39	0.401
Cerebrovascular pathology	2.50	0.80 to 7.80	0.114	1.29	0.66 to 2.52	0.453	2.39	1.17 to 4.89	0.017
Mixed pathology	1.44	0.40 to 5.19	0.575	0.95	0.47 to 1.94	0.892	2.18	1.05 to 4.53	0.036
**Specific MRI markers**									
White matter hyperintensities	1.45	0.92 to 2.31	0.113	1.37	1.02 to 1.85	0.037	2.28	1.65 to 3.15	<0.001
Medial temporal lobe atrophy	1.78	1.01 to 3.14	0.047	1.10	0.77 to 1.57	0.605	1.72	1.19 to 2.48	0.004
Stroke lesion volume^b^	1.00	0.95 to 1.04	0.955	1.01	0.99 to 1.04	0.381	1.02	1.00 to 1.05	0.054
Stroke lesion hemisphere^c^	1.97	0.76 to 5.15	0.165	1.37	0.75 to 2.50	0.308	1.70	0.92 to 3.14	0.091
Stroke lesion location^d^	2.01	0.63 to 6.42	0.235	0.53	0.27 to 1.04	0.065	1.11	0.56 to 2.21	0.760

CI, confidence interval; OR, odds ratio.

Vascular pathology: Pathological white matter hyperintensities score or presence of old infarcts, lacunes or ≥ 2 microbleeds.

Neurodegeneration: Pathological medial temporal lobe atrophy, posterior atrophy, or ventricular enlargement.

Mixed pathology: Presence of both vascular pathology and neurodegeneration.

a Pathology groups were included in analyses as one categorical variable, with normal MRI as reference category.

b *n* = 230.

c *n* = 288, right hemisphere as reference category.

d *n* = 218, white matter as reference category.

**Table 4:** Multinominal logistic regression, one covariate at a time adjusted for age and sex.

Covariate	Impaired cognition			Impaired physical performance			Dual impairment		
	OR	95% CI	*p*-Value	OR	95% CI	*P*-Value	OR	95% CI	*p*-Value
**Pathology groups** ^a^									
Neurodegeneration	1.80	0.49 to 6.66	0.376	0.67	0.27 to 1.64	0.384	1.35	0.51 to 3.61	0.547
Cerebrovascular pathology	1.55	0.47 to 5.10	0.468	0.98	0.48 to 2.00	0.966	1.58	0.71 to 3.51	0.266
Mixed pathology	0.75	0.20 to 2.89	0.678	0.73	0.34 to 1.57	0.423	1.44	0.62 to 3.33	0.396
Age	1.11	1.06 to 1.17	<0.001	1.06	1.03 to 1.09	<0.001	1.06	1.09 to 1.18	<0.001
Male sex	2.49	0.87 to 7.11	0.088	0.81	0.46 to 1.46	0.495	0.84	0.44 to 1.59	0.594
**Specific MRI markers**									
White matter hyperintensities	0.91	0.54 to 1.54	0.725	1.00	0.71 to 1.40	0.993	1.37	0.94 to 1.98	0.098
Age	1.11	1.06 to 1.17	<0.001	1.06	1.03 to 1.10	<0.001	1.13	1.09 to 1.17	<0.001
Male sex	2.63	0.95 to 7.25	0.062	0.79	0.45 to 1.39	0.419	1.04	0.56 to 1.94	0.903
Medial temporal lobe atrophy	1.10	0.58 to 2.08	0.762	0.90	0.60 to 1.36	0.633	1.18	0.76 to 1.82	0.466
Age	1.10	1.05 to 1.17	<0.001	1.06	1.03 to 1.09	<0.001	1.14	1.10 to 1.18	<0.001
Male sex	2.48	0.87 to 7.06	0.088	0.78	0.44 to 1.40	0.408	0.84	0.44 to 1.58	0.581
Stroke lesion volume^b^	1.00	0.96 to 1.05	0.959	1.01	0.99 to 1.04	0.332	1.03	1.00 to 1.05	0.035
Age	1.11	1.05 to 1.17	<0.001	1.06	1.03 to 1.09	<0.001	1.13	1.09 to 1.17	<0.001
Male sex	2.08	1.05 to 1.17	0.204	0.66	0.36 to 1.24	0.196	0.77	0.39 to 1.53	0.459
Stroke lesion hemisphere^c^	1.94	0.72 to 5.22	0.188	1.32	0.70 to 2.48	0.386	1.70	0.87 to 3.34	0.121
Age	1.07	1.01 to 1.13	0.026	1.05	1.02 to 1.09	0.001	1.12	1.08 to 1.17	<0.001
Male sex	1.75	0.50 to 6.16	0.386	0.65	0.32 to 1.29	0.217	0.72	0.33 to 1.54	0.392
Stroke lesion location^d^	2.10	0.64 to 6.83	0.218	0.53	0.26 to 1.06	0.073	1.25	0.58 to 2.70	0.563
Age	1.08	1.01 to 1.14	0.022	1.05	1.02 to 1.09	0.002	1.13	1.08 to 1.18	<0.001
Male sex	1.53	0.43 to 5.44	0.509	0.55	0.27 to 1.12	0.101	0.59	0.27 to 1.30	0.190

CI, confidence interval; OR, odds ratio.

Vascular pathology: Pathological white matter hyperintensities score or presence of old infarcts, lacunes or ≥ 2 microbleeds.

Neurodegeneration: Pathological medial temporal lobe atrophy, posterior atrophy, or ventricular enlargement.

Mixed pathology: Presence of both vascular pathology and neurodegeneration.

a Pathology groups were included in analyses as one categorical variable, with normal MRI as reference category.

b *n* = 230.

c *n* = 288, right hemisphere as reference category.

d *n* = 218, white matter as reference category.

Analyses showed that WMH and MTA were associated with dual impairment—with OR 2.28 (95%CI 1.65 to 3.15), *p* < 0.001, and OR 1.72 (95%CI 1.19 to 2.48), *p* = 0.004, respectively—in the unadjusted analyses (Table 3), but not in the adjusted analyses (Table 4). In contrast, there were no significant associations between the stroke-related variables (lesion location and volume) and impairment group in the unadjusted analysis (Table 3). However, stroke lesion volume became significantly associated with dual impairment after adjusting for age and sex, with OR 1.03 (95% CI 1.00 to 1.05), *p* = 0.035 (Table 4).

## Discussion

In this cross-sectional multicenter cohort study of stroke patients assessed with performance-based tests and MRIs during their initial hospital stay, we found no association between pre-stroke brain pathology and early post-stroke cognitive function or physical performance after adjusting for age and sex. However, among the stroke-specific variables (lesion location in right or left hemisphere/gray or white matter, and volume), a larger stroke lesion volume was associated with dual impairment early after stroke.

The participants with dual impairment were older, had more comorbidities, and had on average suffered a more severe stroke than participants with only one or no impaired domains. These findings share an important contribution with the body of knowledge about the underlying mechanisms of impairments in cognitive and physical function seen after stroke.

Overall, we found a high prevalence of any brain pathology within the whole population (as previously reported in the same study sample) (Schellhorn et al., 2021a), indicating that a considerable proportion of the included participants had substantial brain pathology even before the stroke incident. However, in all four groups of brain pathology, we found heterogeneous presentations of impairments. Almost 40% of participants with no pre-existing brain pathology (normal MRI) had impaired physical performance after their stroke, while between 16 and 22% of participants in the three groups with pathology had no physical or cognitive impairments, indicating no clear pattern between pre-stroke brain pathology and post-stroke impairments. This was confirmed by the adjusted regression analysis.

Even though the findings in the adjusted model were not significant, the cerebrovascular and mixed pathologies had increased odds of 1.58 and 1.44, respectively, for dual impairment, compared to participants with a normal MRI. The association between cerebrovascular pathology, of which WMH is a marker, and dual impairment was supported by the results with MTA and WMH as independent covariates, which showed 37% higher odds (OR 1.37) for dual impairment with a 1-point increase in the WMH burden on the Fazekas scale. The findings on mixed pathology are supported by recent results from a Finnish study showing that a model combining features of vascular pathology and neurodegeneration gave the best prediction of long-term cognitive and functional outcomes in a population with small vessel disease (Jokinen et al., 2021). Moreover, Auriat and colleagues reported that both cognitive and physical outcomes persisting at least 6 months after stroke were associated with white matter changes (Auriat et al., 2019). Our findings of dual impairment in more than one fourth of the study sample indicate that there is a need for targeted rehabilitation to regain and preserve physical performance after stroke. There is substantial evidence of effectiveness of rehabilitative measures for physical performance (Langhorne et al., 2011), while for cognition, the evidence is more diverging (Oberlin et al., 2017). The concurrency of impairments in cognition and physical performance that stroke patients might experience should be considered in rehabilitation settings as especially cognitive function has been found relevant for responsiveness to physical rehabilitation (Lingo VanGilder et al., 2020).

Several previous publications have reported that cerebrovascular pathology, and in particular WMH, is a strong predictor of post-stroke cognitive impairment (Molad et al., 2017; Georgakis et al., 2019; Khan et al., 2019). This was further explored in a subsample of the Nor-COAST population by Aamodt et al., who found that dementia onset within 3 months after stroke was linked to both neurodegenerative and vascular changes (with vascular changes being the most important factor), indicating a mixed etiology (Aamodt et al., 2021). Furthermore, WMH has been reported to be associated with post-stroke physical performance (Sagnier et al., 2020; Dai et al., 2022), indicating an inconsistency in the literature and the need for further research on the importance of vascular pathology (Khan et al., 2019).

Among the stroke-specific variables, only stroke lesion volume was associated with dual impairment after adjustment for age and sex, with 3% greater odds for each milliliter increase in stroke lesion volume; this is in line with findings of infarct volume being the strongest predictor of motor and cognitive impairments after stroke (Zaidi et al., 2012; Helenius and Henninger, 2015; Schellhorn et al., 2021b). With regard to physical performance, stroke lesion volume has been reported to be associated with balance and gait recovery, and basal ganglia involvement seems to be a negative factor for gait speed at one-year post-stroke (Nadeau et al., 2016). This, together with the role of pre-existing pathology, is an argument for both focal and global brain pathology contributing to this clinical presentation.

Even though the finding was not significant, a lesion in the left hemisphere almost doubled the odds of cognitive impairment (OR 1.94) and increased the odds of dual impairment by 70% (OR 1.7) compared to lesions in the right hemisphere, while lesions in the gray matter increased the risk for cognitive impairment by 25% compared to white matter. In stroke-free individuals, specific areas of gray matter have been identified as playing critical roles in both cognition and physical performance; individuals with dual impairment had more atrophy in these regions than the general population (Tian et al., 2021), which could be a possible underlying explanation of our findings. With regard to hemisphere, previous findings are contradictory, as one study reported left hemispheric stroke as a risk factor of post-stroke cognitive impairment (Sagnier et al., 2019), while others have found no differences between hemispheres in either cognition (Puy et al., 2018) or physical performance (Nadeau et al., 2016). The lack of significant associations might be caused by lack of power due to the dichotomization of location into white or gray matter, as post-stroke cognitive impairment has been associated with various more-specific strategic locations (Weaver et al., 2021). Hence, future research is needed to confirm these associations.

On average, the dual impairment group was older, had a higher burden of comorbidity, and had more functional dependency compared to the other impairment groups. This is in line with findings from previous publications that have looked at dual decline in cognition and physical performance over time (Beauchet et al., 2020; Tian et al., 2020). The reduction in cognition and physical performance might be an expression of reduced brain resilience that already existed before the stroke; additionally, dual impairment could be an expression of frailty, which has previously been shown to be associated with reduced quality of life after stroke (Wæhler et al., 2021).

MRIs during the acute stroke phase might contribute to early identification of stroke patients at risk of dual impairment in cognition and physical performance by combining information from pre-existing and stroke-specific brain pathology; this could, in turn, lead to actions preventing further functional decline. Further research should investigate the extent to which a model based on these variables predicts function at 3 or 18 months post-stroke. Pre-stroke imaging characteristics did not reach significant associations with post-stroke impairment in our study, indicating that stroke lesion characteristics are more important than pre-stroke pathology for presence of post-stroke dual impairment.

### Strengths and limitations

The present study has several strengths and some limitations that need to be addressed. The study is based on a uniquely large dataset that includes clinical assessments and study-specific MRI at the time of the indexed stroke. However, the pre-existing pathology was only assessed by visually rating, which is commonly done in clinical settings, and not quantitative measures which the stroke lesion characteristics were assessed with. Another strength is the use of the SPPB and MoCA, two thoroughly validated clinical assessments that are frequently applied in clinical and research settings. Although the cut-offs applied for the MoCA and SPPB are validated, they were not adjusted for age, sex, or education, which could have led to an overestimation of impairments in the oldest participants. Our method of classifying multiple strokes into white or gray matter lesions has not been validated. Even though this should be regarded as a weakness, it is unlikely that it has affected our results as few cases suffered from stroke both in gray and white matter. In addition, the small sample size, especially in the group with impaired cognition, inhibited us from adding more covariates (e.g., education) to the regression analyses. Further, the dichotomization of the SPPB and MoCA variables could possibly have led to loss of statistical power. Age but not sex was significantly associated with post-stroke impairment outcomes in the adjusted analyses. This is expected as both cerebral pathology and performance on cognitive and physical assessments worsens with age. Regarding cognitive status, we did not have information on amyloid burden, which would have been relevant for assessing etiology of cognitive impairment. And lastly, information about pre-stroke function as measured by mRS was collected retrospectively at time of stroke. More details about pre-stroke physical performance and cognitive function would also have been a strength, as the stroke in itself increases risk of cognitive and physical impairments even after adjusting for pre-stroke factors (Einstad et al., 2022).

Overall, the Nor-COAST population has shown to be comparable to the majority of the Norwegian stroke population suffering from mild to moderate strokes (Kuvås et al., 2020). From the original Nor-COAST cohort, 43% of the participants were available for inclusion in the present study; those lost to follow-up were significantly older and had suffered larger strokes. As a result, the results from this study can only be generalized to the healthier members of the stroke population (Kuvås et al., 2020). The cross-sectional design limits us from drawing any conclusions on causality, and the dichotomization of clinical and imaging variables might have led to underestimation of statistical significance.

### Conclusion

The overall findings from the present study include the lack of significant associations with pre-stroke brain pathology and the significant association between larger stroke volume and dual impairment of cognitive and physical performance early after stroke (after adjustment for age and sex). The study also indicated that WMH and stroke lesion location in the left hemisphere were associated with dual impairment, although these findings were not significant. This study also showed that dual impairment is a rather common problem after stroke; however, the etiology of this phenotype should be further investigated. Future studies should also include longitudinal designs exploring the changes over time in post-stroke physical performance and cognition in relation to brain pathology. Such knowledge will be important when designing new and more individualized rehabilitation interventions after strokes.

## Data availability statement

The datasets presented in this article are not readily available due to Norwegian regulations and conditions for informed consent. Requests to access the datasets should be directed to IS, ingvild.saltvedt@ntnu.no.

## Ethics statement

The studies involving human participants were reviewed and approved by Regional Committee for Medical and Health Research Ethics (REC Central 194265). The patients/participants provided their written informed consent to participate in this study.

## Author contributions

IS is the primary investigator of the Nor-COAST study. IS, PT, TA, and ME developed the design of the present study. ME, TA, and SL were responsible for the analysis plan. ME and SL planned the statistical analyses and ME performed them. ME and TA were the responsible for the writing of the present report. EA and TS did the data curation of MRI variables. TS and MB performed the visual analyses of images. All authors interpreted the results, read, and approved of the final manuscript.

## Funding

The Nor-COAST study is funded by the Norwegian Health Association, and additional funding was provided by the Department of Neuromedicine and Movement Science, Faculty of Medicine and Health Science, NTNU-Norwegian University of Science and Technology. Open access publication fees were generously funded by the Norwegian University of Science and Technology.

## Conflict of interest

The authors declare that the research was conducted in the absence of any commercial or financial relationships that could be construed as a potential conflict of interest.

## Publisher’s note

All claims expressed in this article are solely those of the authors and do not necessarily represent those of their affiliated organizations, or those of the publisher, the editors and the reviewers. Any product that may be evaluated in this article, or claim that may be made by its manufacturer, is not guaranteed or endorsed by the publisher.
